# Supplementary material for: Strategies to Improve the Properties of Amaranth Protein Isolate-Based Thin Films for Food Packaging Applications: Nano-Layering through Spin-Coating and Incorporation of Cellulose Nanocrystals
Source: Nanomaterials (Basel). 2020 Dec 21;10(12):2564. doi: 10.3390/nano10122564 (PMC7766300; doi:10.3390/nano10122564)
Supplement: Supplementary file 1 [file nanomaterials-10-02564-s001.pdf]

nanomaterials

SUPPLEMENTARY MATERIAL

Article

Strategies to Improve the Properties of Amaranth Protein Isolate-Based Thin Films for Food Packaging Applications: Nano-layering through Spin-Coating and Incorporation of Cellulose Nanocrystals

Amparo López-Rubio, Adriana Blanco-Padilla, Kristiina Oksman and Sandra Mendoza

SEM analysis of the nanocomposite films

The microstructure of the nanocomposite films was studied by means of Scanning Electron Microscopy (Hitachi S-4800). Films were cryo-fracturated after immersion in liquid nitrogen and randomly broken to investigate the cross-section of the samples. Cryo-fractured films were mounted on aluminium stubs and fixed on the support using a double-size adhesive tape. Finally, samples were gold–palladium coated and observed using an accelerating voltage of 10kV and a working distance of 10 mm.

Figure S1 shows the cross-sections of pure API and the various CNC-containing API nanocomposite films. As it can be inferred from the micrographs, no visual CNC agglomeration could be observed and rather homogeneous cross-sections were obtained independently of the CNC content, thus highlighting the excellent compatibility between the API matrix and the nanofiller.

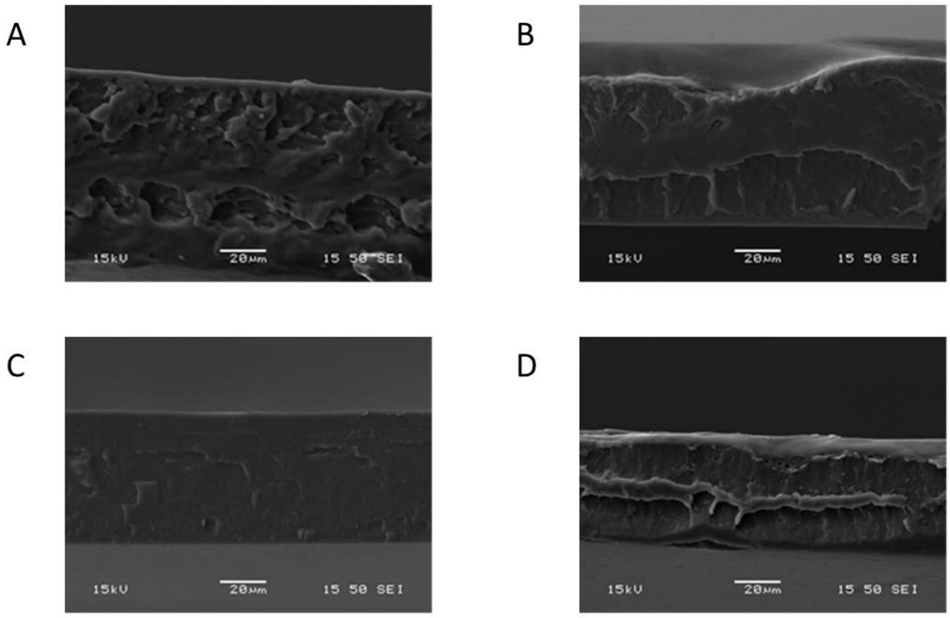

Figure S1. SEM images of the cross-sections of: (A) API; (B) API with 5% CNC; (C) API with 10% CNC; (D) API with 20% CNC
